# Pharmacokinetics, safety and tolerability of ipatasertib in combination with palbociclib and fulvestrant in patients with advanced breast cancer in a phase Ib study

**DOI:** 10.3389/fphar.2025.1670582

**Published:** 2025-09-26

**Authors:** Kit Wun Kathy Cheung, Ksenia Arzumanova, Victor Poon, Adam Harris, Ryan Johnson, Frauke Schimmoller, Rucha Sane

**Affiliations:** Genentech, Inc., South San Francisco, CA, United States

**Keywords:** ipatasertib, breast cancer, clinical study, drug-drug interactions, cyp3a

## Abstract

**Introduction:**

Ipatasertib is a potent, highly selective, small molecule AKT inhibitor that has been evaluated in combination with palbociclib and fulvestrant for the treatment of hormone receptor-positive (HR+) human epidermal growth factor receptor two negative (HER2-) breast cancer. Ipatasertib is a sensitive CYP3A4 substrate and is extensively metabolized to its major metabolite, M1 (G-037720). Ipatasertib is also a mild to moderate CYP3A inhibitor *in vitro*. Palbociclib is a weak time-dependent CYP3A inhibitor and a CYP3A substrate. Accordingly, drug-drug interaction (DDI) between ipatasertib and palbociclib is expected when the two drugs are co-administered.

**Methods:**

The study reported herein is a Phase Ib clinical trial that aimed to evaluate the safety and pharmacokinetics (PK) of 300 mg ipatasertib in combination with palbociclib and fulvestrant (NCT04060862). The ipatasertib and M1 PK at steady state as a single agent were compared to that in combination with palbociclib and fulvestrant to evaluate the magnitude of DDI between ipatasertib and palbociclib.

**Results:**

The PK analysis showed that the area under the concentration-time curve from time 0–24 h at steady state (AUC_0-24,ss_) and the maximum observed plasma concentration at steady state (C_max,ss_) of ipatasertib increased by 68% and 49%, respectively, when ipatasertib was coadministered with palbociclib and fulvestrant compared to administration of ipatasertib alone. A similar trend was observed for M1 with AUC_0-24,ss_ and C_max,ss_ increased by 20% and 14%, respectively, when ipatasertib was coadministered with palbociclib and fulvestrant compared to administration of ipatasertib alone. Palbociclib plasma trough concentrations at steady state were generally comparable with historical data.

**Conclusion:**

This study indicated a DDI between ipatasertib and palbociclib, leading to increased ipatasertib exposure. The combination regimen of ipatasertib 300 mg with palbociclib and fulvestrant had a notable and manageable safety profile, that is generally consistent with the known risks of each individual study drugs in patients with HR + HER2-breast cancer.

## Introduction

Breast cancer is the most frequently diagnosed cancer in women, with over 2.3 million new cases reported in 2020 (Arnold et al., 2022). Hormone receptor-positive (HR+) human epidermal growth factor receptor two negative (HER2-) breast cancer accounts for approximately 70% of all breast cancer subtypes (Fedele et al., 2018). Endocrine therapy, which includes selective estrogen receptor modulator, selective estrogen receptor down-regulators (SERDs) and aromatase inhibitors (AIs), is recommended alone or in combination with targeted therapy for HR + HER2-advanced or metastatic breast cancer, unless in visceral crisis when chemotherapy is indicated, according to clinical guidelines (Ngan, 2018). CDK4/6 inhibitors, such as palbociclib, have been approved as both first-line and second-line treatments in HR + HER2-patients, and have been shown to significantly improve efficacy over endocrine therapy alone (Finn et al., 2015; Cristofanilli et al., 2016; Hortobagyi et al., 2016; Goetz et al., 2017; Sledge Jr et al., 2017).

Despite efforts to enhance the clinical benefit of endocrine therapy, many patients experience refractory disease and poor responses due to high endocrine resistance. Various mechanisms contributing to primary and/or secondary endocrine resistance in HR + breast cancer have been identified. One such mechanism involves alterations in the PI3K/AKT/mTOR pathways, which are observed in approximately 50% of HR + HER2-breast cancers (Johnston, 2009; Musgrove and Sutherland, 2009; Koboldt et al., 2012; Robinson et al., 2013; Toy et al., 2013; Jeselsohn et al., 2014). Combining an AKT inhibitor with fulvestrant has demonstrated greater efficacy compared to fulvestrant alone (Howell et al., 2022; Turner et al., 2022).

Ipatasertib is a potent, highly selective, small-molecule inhibitor of all three isoforms of the serine/threonine kinase AKT (Saura et al., 2017). It has been developed as a single agent and in combination with other therapies for treating cancers where activation of the PI3K-AKT-mTOR pathway may be relevant to tumor growth or therapeutic resistance. The Phase Ib study reported herein (NCT04060862) aimed to evaluate the combination of ipatasertib, palbociclib and fulvestrant in patients with HR + HER2-locally advanced unresectable or metastatic breast cancer. Other studies where ipatasertib (400 mg) was administered in combination with endocrine therapy included the Phase III FINER study (NCT04650581), Phase II FAIM study (NCT04920708) and Phase I TAKTIC trial (NCT03959891).

Ipatasertib exposure was found to be dose-proportional over the range of 200–800 mg. The effective half-life of ipatasertib is approximately 24 h. Dedicated food effect study suggested that ipatasertib can be administered with or without food (Malhi et al., 2021). Ipatasertib is a sensitive substrate of cytochrome P450 3A4 (CYP3A4) and is extensively metabolized by this enzyme to its major and pharmacologically active metabolite, M1 (G-037720) (Sane et al., 2021). Additionally, ipatasertib is a mild to moderate CYP3A inhibitor *in vitro* (Malhi et al., 2021). Palbociclib, on the other hand, is a weak time-dependent CYP3A inhibitor and a CYP3A substrate (U.S. Food and Drug Administration, 2023). Consequently, a drug-drug interaction (DDI) between ipatasertib and palbociclib was anticipated and a 300 mg dose, which is lower than previously employed 400 mg dose, was used in this combination study to achieve appropriate level of ipatasertib exposures. The current study investigated the impact of palbociclib on the exposure of ipatasertib and M1. This manuscript presents the pharmacokinetic (PK) and safety data of ipatasertib both in the presence and absence of palbociclib from this Phase Ib study.

## Methods

### Study design and treatment

**FIGURE 1:**
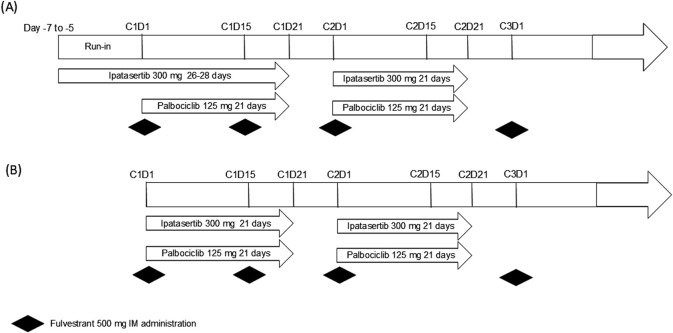
Study design for the Phase Ib study **(A)** with ipatasertib run-in (n = 10) and **(B)** the additional 10 patients for further safety evaluation.

The study design for the open-labeled Phase Ib study is shown in Figure 1. The study initially enrolled 10 patients, who received a starting dose of 300 mg ipatasertib orally once daily (QD) for 5–7 days (referred to as “ipatasertib run-in”) as a single agent (Figure 1A). This dose was lower than the intended therapeutic dose of 400 mg that was administered in other ipatasertib clinical studies to mitigate the potential increase in ipatasertib exposure upon combining with palbociclib. Given the half-life of ipatasertib, the run-in of 5–7 days allows the steady state to be reached prior to PK sample collection. This phase preceded the initiation of palbociclib and fulvestrant on Day 1 of Cycle 1. Hence, for the first cycle, ipatasertib was administered continuously for 26–28 days, starting from Day −7 to Day −5 window of the initial single-agent ipatasertib run-in. From Cycle 2 Day 1 onward, ipatasertib was taken orally and daily on Days 1–21 of each 28-day cycle. Starting on Day 1 of Cycle 1, palbociclib 125 mg capsule was administered orally daily on Days 1–21 of each 28-day cycle. Per prescribing information, palbociclib capsules were taken with food to reduce the intersubject variability of palbociclib exposure (U.S. Food and Drug Administration, 2023). Additionally, fulvestrant 500 mg was given via intramuscular (IM) injection on Days 1 and 15 of Cycle 1, and then on Day 1 of each subsequent 28-day cycle. Of note, on Day 1 of Cycle 1, both palbociclib and fulvestrant were administered approximately 6 h after the administration of ipatasertib to enable collection of post-dose PK samples of ipatasertib. Starting from Day 2 of Cycle 1, palbociclib would be taken at the same time as ipatasertib. As ipatasertib can be administered with or without food, the coadministration of palbociclib and ipatasertib in the presence of food was acceptable. The initial safety follow-up extended through at least Day 15 of Cycle two for all patients. To further evaluate the safety of the combination, an additional 10 patients were enrolled and were received dosing without undergoing the run-in phase (Figure 1B). These additional patients started all three study drugs on Day 1 of Cycle 1.

Use of strong CYP3A inhibitors, strong CYP3A inducers and sensitive CYP3A substrates with narrow therapeutic window were prohibited within 14 days (or five drug-elimination half-lives, whichever is longer) prior to and during the study treatment period and for 7 days after the last dose of study treatment.

PK samples for ipatasertib and its major metabolite, M1 (G-037720), were collected at specified time points: 15 min pre-dose, 0.5, 1, 2, 3, 4, 6 h post-dose on Day 1 of Cycle 1, and Day 15 of Cycle 1, as well as 15 min pre-dose on Day 15 of Cycle 2 and 15 min pre-dose and 2 h post-dose on Day 15 of Cycle 3. PK samples for palbociclib were collected 15 min pre-dose on Day 15 of Cycles 1 to 3.

### PK analysis

The plasma concentrations of ipatasertib and its metabolite M1 was determined using a previously reported, validated liquid chromatography-tandem mass spectrometry assay (LC-MS/MS) (Sutaria et al., 2022). Plasma concentrations of palbociclib were determined using a validated LC-MS/MS assay in accordance with the 2018 FDA Bioanalytical Method Validation Guidance (U.S. Food and Drug Administration, 2018). The sample analyses were performed at Labcorp Early Drug Development Laboratories, Inc. (Madison, WI, Unitd States) using a Shimadzu LC instrument equipped with an Agilent Polaris C8-A column (2.0 × 50 mm, 5 μm) interfaced with a Sciex API 4000 mass spectrometer operated in positive electrospray ionization mode. Gradient elution chromatography utilizing 0.1% formic acid in water (mobile phase A) and 0.1% formic acid in acetonitrile (mobile phase B) was used to separate palbociclib from matrix components. The MS/MS transitions monitored for the assay were m/z 448.0 to 380.0 for palbociclib and 456.0 to 388.0 for the stable-labeled internal standard palbociclib d-8. The linear dynamic range of the assay was 5 to 1,000 ng/mL palbociclib, and dilutional integrity was validated for samples containing up to 5,000 ng/mL. All study samples were analyzed within the established long-term frozen stability of palbociclib in human plasma.

Noncompartmental PK analysis (NCA) was performed using the commercial software Phoenix WinNonlin (Certara Unitd States, Inc., Princeton, NJ, Unitd States, Version 8.1) to characterize the PK parameters of ipatasertib and M1 including maximal concentration (C_max_), time to maximal concentration (T_max_), area under the curve from time 0 to the last measurable concentration (AUC_0-t_). For palbociclib, PK parameters [AUC, C_max_ and trough concentration (C_min_)] were estimated using an established population PK (popPK) model (U.S. Food and Drug Administration, 2014) and were compared to available historical and literature data.

### Safety assessment

Safety assessments consisted of monitoring and recording adverse events, including serious adverse events and adverse events of special interest, performing protocol-specified safety laboratory assessments, measuring protocol-specified vital signs, and conducting other protocol-specified tests that are deemed critical to the safety evaluation of the study. The adverse event severity grading scale for the NCI Common Terminology Criteria for Adverse Events (CTCAE) v5.0 was used for assessing adverse event severity. If daily dosing of ipatasertib was not tolerated, dosing with food might be used to alleviate gastrointestinal symptoms. To manage drug-related toxicities, dose modifications for ipatasertib and palbociclib were pre-specified. For ipatasertib, with a starting dose of 300 mg, the first dose reduction was to 200 mg and the second down to 100 mg. For palbociclib, with a starting dose of 125 mg, the first dose reduction was down to 100 mg and the second down to 75 mg. Dose re-escalation of ipatasertib and palbociclib were not permitted.

### Statistical analysis

Non-compartmental PK analyses were performed using Phoenix(R) WinNonlin (Version 8.3, Certara). Descriptive statistics (number of patients, mean, SD, %CV, median, min, max, geometric mean, and geometric %CV) were summarized for PK parameters for ipatasertib and M1 (G-037720). Appropriate PK parameters (area under the curve from time 0–24 h at steady state [AUC_0-24,ss_], maximal concentration at steady state [C_max,ss_]) from Day 1 of Cycle one (ipatasertib alone) and Day 15 of Cycle one (ipatasertib + palbociclib + fulvestrant) were compared to assess the effect of palbociclib on the PK of ipatasertib by calculating the geometric mean ratios and the corresponding 90% confidence intervals (CIs) to evaluate the effect of palbociclib on the PK of ipatasertib and M1 (G-037720). A linear mixed model with a fixed effect for treatment and a random effect for patients was used on the natural log-transformed PK parameters. Point estimates for the means and point estimates and corresponding 90% CIs for the differences in means between the two treatments (reference and test treatments) were obtained from the linear mixed effects model and then exponentiated to obtain geometric means, geometric mean ratios, and respective 90% CIs on the original scale.

## Results

### Subject demographics

Twenty female patients were enrolled in this study with a median age of 55 years (range: 37–74 years) (Table 1). The majority (75%, n = 15) of the enrolled patients were white. Sixty-five percent of the patients had primary endocrine resistance with relapse less than 2 years after starting adjuvant endocrine therapy and 80% had received prior neoadjuvant or adjuvant chemotherapy. All patients had an European Cooperative Oncology Group (ECOG) grade of 0 or 1.

**TABLE 1:** Summary of demographics and baseline characteristics of patients enrolled in this study.

Characteristics	Category/Statistics	Number of patients (%)
Age (years)	Median (range)	55.0 (37–74)
Sex, n (%)	Female	20 (100)
Race, n (%)	White	15 (75)
	Asian	4 (20)
	Black	1 (5)
ECOG performance status, n (%)	0	15 (75)
	1	5 (25)
Prior (neo)adjuvant chemotherapy, n (%)		16 (80)
Prior adjuvant endocrine therapy, n (%)	Tamoxifen	9 (45)
	Aromatase inhibitor	13 (65)
Duration of last adjuvant endocrine therapy (primary/secondary resistance), n (%)	≤2 years	13 (65)
	>2 years	7 (35)
Metastatic disease at study enrollment, n (%)		20 (100)
Visceral metastases, n (%)	Lung and/or liver	12 (60)
	Liver	9 (45)
	Lung	7 (35)
Time since initial diagnosis, years	Median (range)	4.3 (1.2–10.7)

### PK analysis

The impact of palbociclib on the exposure of ipatasertib and M1 (G-037720) were assessed based on data from nine out of the 10 patients who were enrolled in the initial phase with ipatasertib run-in (Figure 1A). One patient was excluded from the analysis due to a dose reduction prior to PK evaluation. Additionally, plasma concentrations of palbociclib, following multiple combination dosing with ipatasertib and fulvestrant, were analyzed and are reported for all enrolled patients.

#### Ipatasertib pharmacokinetics in the presence and absence of palbociclib

**FIGURE 2:**
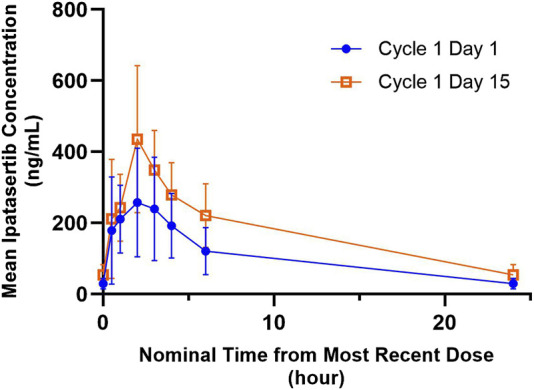
Arithmetic mean ± SD concentration-time profiles of ipatasertib following multiple doses of orally administered ipatasertib 300 mg QD alone and in combination with palbociclib and fulvestrant.

The mean ipatasertib plasma concentration *versus* time profiles for 300 mg QD ipatasertib alone and in combination with 125 mg palbociclib and fulvestrant are presented in Figure 2. Specifically, the AUC_0-24,ss_ increased by 1.68-fold (90% CI 1.41–2.00) and the C_max,ss_ increased by 1.49-fold (90% CI 1.18–1.88) (Table 2).

**TABLE 2:** Summary of ipatasertib and M1 (G-037720) pharmacokinetics following multiple dose administration of ipatasertib (300 mg) QD alone on Day 1 of Cycle 1 and in combination with palbociclib and fulvestrant on Day 15 of Cycle 1.

Analyte	PK parameter	C1D1 Ipatasertib alone	C1D15 Ipatasetib in combination with palbociclib and fulvestrant	Geometric mean ratio (GMR) (90% CI)
		N = 9^a^	N = 9^a^	
Ipatasertib	AUC_0-24,ss_ (ng*hr/mL)	2,169.9 (46.3%)	3,637.0 (33.7%)	1.68 (1.41, 2.00)
	C_max,ss_ (ng/mL)	293.8 (52.6%)	437.3 (41.1%)	1.49 (1.18, 1.88)
	T_max_ (hr)	1.0 (0.5–4.0)	1.9 (0.5–4.0)	NA
M1 (G-037720)	AUC_0-24,ss_ (ng*hr/mL)	1157.0 (76.6%)	1391.6 (44.9%)	1.20 (0.99, 1.46)
	C_max,ss_ (ng/mL)	120.1 (84.2%)	137.4 (53.4%)	1.14 (0.86, 1.52)
	T_max_ (hr)	2.0 (1.0–4.0)	2.0 (1.0–3.1)	NA

AUC, area under the concentration-time curve; AUC_0-24,ss_ = AUC from time 0–24 h at steady state; C1D1 = Day 1 of Cycle 1; C1D15 = Day 15 of Cycle 1; C_max,ss_ = maximum observed plasma concentration at steady state; CI, confidence interval; GMR, Geometric Mean Ratio of exposure at C1D15 (test; ipatasertib in presence of palbociclib and fulvestrant)/C1D1 (reference; ipatasertib alone); NA, not applicable; T_max_ = time to maximum concentration.

^a^ One subject was excluded from this analysis due to ipatasertib dose reduction prior to PK evaluation.

Note: All participants included in these analyses were treated with ipatasertib 300 mg for 5–7 days to reach steady state as a single agent before palbociclib and fulvestrant started on Day 1 of Cycle 1 after single agent PK sampling was completed. All parameters are reported as geometric mean (%geoCV), except T_max_, which is reported as median (range).

#### M1 (G-037720) pharmacokinetics in the presence and absence of palbociclib

**FIGURE 3:**
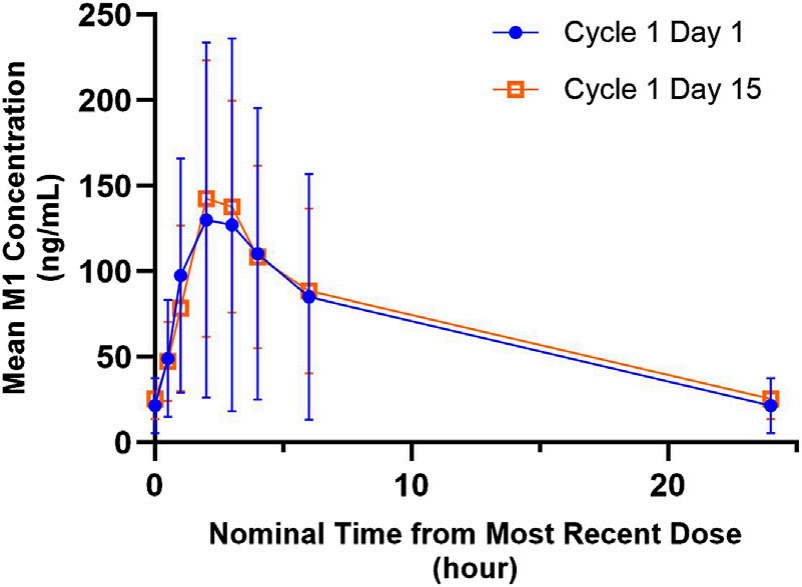
Arithmetic mean ± SD concentration-time profiles of M1 (G-037720) following multiple doses of orally administered ipatasertib 300 mg QD alone and in combination with palbociclib and fulvestrant.

The mean M1 (G-037720) plasma concentration *versus* time profiles for 300 mg QD ipatasertib, both as monotherapy and in combination with 125 mg palbociclib and fulvestrant, are presented in Figure 3. The GMR (90% CI) for AUC_0-24,ss_ and C_max,ss_ of M1 (G-037720) were 1.20 (0.99–1.46) and 1.14 (0.86–1.52), respectively (Table 2).

#### Palbociclib pharmacokinetics following multiple combination dosing with ipatasertib and fulvestrant

A summary of plasma concentrations of palbociclib at specific predose (C_min,ss_) timepoints following multiple combination dosing of ipatasertib, palbociclib and fulvestrant is shown in Table 3. The plasma trough concentrations of palbociclib were generally comparable across all the timepoints and between the initial 10 patients and the subsequent 10 patients enrolled in the study for additional safety evaluation. Palbociclib PK parameters were estimated using an established popPK model (U.S. Food and Drug Administration, 2014); the geometric mean (%CV) for AUC_0-24,ss_, C_max,ss_ and C_min,ss_ were 2,596 ng*hr/mL (24.1%), 124 ng/mL (20.9%) and 87.0 (30.9%), respectively.

**TABLE 3:** Summary of palbociclib plasma concentrations at nominal predose timepoints following administration of ipatasertib in combination with palbociclib and fulvestrant.

Analyte	Dose (mg)	Group	Statistic	C1D15 predose	C2D15 predose	C3D15 predose
Palbociclib	125	Ipatasertib run-in^a^	N	9	10	10
			Geomean (ng/mL) (geoCV%)	69.9 (57.2)	76.9 (44.9)	71.5 (43.5)
		Additional patients for safety evaluation^b^	N	9	6	9
			Geomean (ng/mL) (geoCV%)	73.8 (20.7)	83.2 (19.4)	67.5 (51.0)
		Total	N	18	16	19
			Geomean (ng/mL) (geoCV%)	71.8 (40.8)	79.2 (36.4)	69.6 (45.7)

C = cycle; D = day; geoCV% = geometric mean coefficient of variations; Geomean = geometric mean; N = number of participants.

^a^ First 10 participants in run-in were treated with ipatasertib 300 mg for 5–7 days as a single agent before palbociclib and fulvestrant started on Day 1 of Cycle 1.

^b^ Additional 10 participants started ipatasertib, palbociclib and fulvestrant on Day 1 of Cycle 1.

### Summary of adverse events

Grade 3 and 4 adverse events (AEs) occurred in 17 of 20 (85%) of patients. Among these AEs, the most frequently reported by CTCAE preferred terms (PT) were neutropenia (8 patients), neutrophil count decreased (7 patients) and white blood cell count decreased (4 patients).

Overall, the most frequent reported AEs of any grade were diarrhea (85%), nausea (80%), constipation and vomiting (50%), neutropenia (45%) and asthenia, rash, anaemia and neutrophil count decreased (40% each).

When attributed to individual drugs, the most frequent AEs considered related to ipatasertib included diarrhea (75%), nausea (70%) and vomiting (45%). Other AEs related to ipatasertib included rash (35%); anemia, neutropenia and platelet count decreased (30% each); and fatigue and neutrophil count decreased (25% each). The most frequent AEs considered related to palbociclib were neutropenia (45%); nausea, anemia, and neutrophil count decreased (40% each); diarrhea and platelet count decreased (30% each); and stomatitis and asthenia (25% each). For fulvestrant, the most frequent AEs related AEs were asthenia (15%) and anemia and hot flush (10%). Overall, the types of AEs attributed to each drug are consistent with their known, individual safety profiles. An overview of the safety events is reported in Table 4.

**TABLE 4:** Combined summary of adverse events for all enrolled patients.

AE category	Number of patients (%)
Total number of patients with at least one AE	20 (100%)
Total number of AEs	446
Total number of deaths	0
Total number of patients with at least one	
Any treatment discontinuation	1 (5%)
AE leading to discontinuation of ipatasertib	1 (5%)
AE leading to discontinuation of palbociclib	1 (5%)
AE leading to discontinuation of fulvestrant	0
AE leading to dose reduction of ipatasertib	9 (45%)
AE leading to dose reduction of palbociclib	10 (50%)
AE leading to dose reduction of fulvestrant	0
AE leading to dose interruption of ipatasertib	16 (80%)
AE leading to dose interruption of palbociclib	13 (65%)
AE leading to dose interruption of fulvestrant	5 (25%)
Grade ≥ 3 AE	17 (85.0%)
Grade 5 AE	0
Serious AE	4 (20.0%)
AE related to any treatment	20 (100%)
AE related to ipatasertib	20 (100%)
AE related to palbociclib	20 (100%)
AE related to fulvestrant	16 (80%)

Multiple occurrences of the same AE in one individual are counted only once except for “Total number of AEs” row in which multiple occurrences of the same AEs are counted separately.

## Discussion

During drug development, a critical aspect to consider when evaluating the combinability of therapeutic agents is the potential for DDI between the agents. DDIs may not be inherently detrimental; for instance, DDIs have been utilized to enhance the exposure of certain antiretroviral agents that are CYP3A substrates when combined with ritonavir or cobicistat, both of which strongly inhibit CYP3A (Kaur et al., 2015; Yu et al., 2023). When the magnitude of the DDI is well characterized, dosing adjustment can be made to normalize the exposure of the victim drug in the presence of the perpetrator drug.

In this study, a DDI between ipatasertib and palbociclib was anticipated based on the known metabolic profiles and DDI liabilities of the two agents. While the small sample size for the DDI portion of the study (n = 9) is a limitation, the study design was strengthened by the self-controlled PK analysis and the enrollment of additional patients with a total sample size of 20, expanding the characterization of the regimen’s tolerability. Data from previous DDI studies were leveraged to quantitatively estimate the extent of interaction prior to the initiation of this study. Ipatasertib is a sensitive CYP3A substrate; when given as a 100 mg single dose with itraconazole, a strong CYP3A4 and P-gp inhibitor, the area under the curve from time 0 to infinity (AUC_0-∞_) and maximum concentration (C_max_) of ipatasertib increased by 5.45-fold and 2.26-fold, respectively (Sane et al., 2021). Palbociclib is a mild CYP3A inhibitor, which increased the AUC_0-∞_ and C_max_ of midazolam by 61% and 37%, respectively (U.S. Food and Drug Administration, 2023). Consequently, palbociclib was expected to increase the exposure of ipatasertib to a similar magnitude when given concurrently.

Using a physiologically-based pharmacokinetic modeling approach, the extent of this DDI was simulated, with the predicted increase in the AUC and C_max_ of ipatasertib 300 mg being 48% and 23%, respectively, in the presence of palbociclib (Jing et al., 2022). Based on this expected DDI, the dose of ipatasertib tested in this study was lowered by 25%–300 mg from the intended therapeutic dose of 400 mg that was administered in other clinical studies to accommodate the potential increase in ipatasertib exposures to safely coadminister the two drugs (Dent et al., 2021; Turner et al., 2022). Our results demonstrated that the AUC_0-24,ss_ and C_max,ss_ of ipatasertib increased by 68% and 49%, respectively, in the presence of palbociclib compared to when ipatasertib was given alone. Furthermore, the AUC_0-24,ss_ of ipatasertib at 300 mg in the presence of palbociclib was about 1.25 times higher than that at 400 mg in other clinical studies (3,637 ng*hr/mL vs. 2,920 ng*hr/mL) (Yoshida et al., 2021). Overall, the increase in ipatasertib exposure in the presence of palbociclib was consistent with that simulated by PBPK.

A similar trend was observed for M1 (G-037720) when ipatasertib was coadministered with palbociclib and fulvestrant compared to when ipatasertib was administered alone. The C_max,ss_ and AUC_0-24,ss_ increased marginally by 14% and 20%, respectively. M1 was formed and subsequently metabolized by CYP3A4, as determined by *in vitro* studies with human liver microsomes (Takahashi et al., 2023). Given that M1 demonstrates formation rate limited kinetics, the slight increase in M1 exposure can plausibly be explained by a combination effect of reduction in formation as well as elimination (Sane et al., 2021).

Ipatasertib is characterized as a mild to moderate CYP3A4 inhibitor *in vitro* (Malhi et al., 2021). When a single dose of ipatasertib 600 mg was administered with midazolam, the AUC_0-_ ∞ of midazolam increased by 2.22-fold (Malhi et al., 2021). Palbociclib is primarily metabolized by CYP3A and SULT2A1 (Yu et al., 2017; U.S. Food and Drug Administration, 2023). When administered with multiple doses of itraconazole, the C_max_ and AUC_0-∞_ of palbociclib increased by 34% and 87%, respectively, compared to when it was administered alone, indicating that palbociclib is not a sensitive substrate of CYP3A4. The C_min_ of palbociclib reported in this study (approximately 70 ng/mL; Table 3) were comparable to the historical levels previously reported when palbociclib was administered with fulvestrant, where the geometric mean C_min,ss_ ranged from 74.8 ng/mL to 86.3 ng/mL (Masuda et al., 2019). Further the popPK-estimated C_max,ss_ (124 ng/mL) and AUC_0-24,ss_ (2,596 ng*hr/mL) of palbociclib in this study were also comparable to that when palbociclib was administered alone (116 ng/mL and 1982 ng*hr/mL for C_max,ss_ and AUC_0-24,ss_, respectively) (U.S. Food and Drug Administration, 2014). In line with the dosing guidance of palbociclib, dose adjustment for palbociclib is not warranted when co-administered with ipatasertib, given the mild interaction.

The DDI assessment focused on the interaction between ipatasertib and palbociclib within the triplet combination with fulvestrant. Fulvestrant is not an inhibitor or inducer of CYP3A and therefore is not expected to alter the exposure of ipatasertib and palbociclib (U.S. Food and Drug Administration, 2021). Further, fulvestrant is a CYP3A substrate *in vitro*, but strong CYP3A inhibitors and inducers did not have clinically relevant impact on fulvestrant disposition (U.S. Food and Drug Administration, 2021). Therefore, ipatasertib is not expected to alter the exposure of fulvestrant in a clinically meaningful manner.

The safety profile of ipatasertib in combination with palbociclib and fulvestrant was generally consistent with the known risks of each individual study treatment component, and no new safety signals were identified (Dent et al., 2021; U.S. Food and Drug Administration, 2021; U.S. Food and Drug Administration, 2023; Turner et al., 2022). For instance, in this study 75% of patients had diarrhea that were attributed to ipatasertib. In the LOTUS study, a double-blind placebo controlled randomized Phase 2 study of first-line ipatasertib plus paclitaxel for inoperable locally advanced/metastatic triple-negative breast cancer, 93% of patients that received the ipatasertib-paclitaxel experienced any grade of diarrhea as compared to 21% of those that received placebo-paclitaxel (Dent et al., 2021). While 85% of patients experienced Grade 3 or 4 AEs, these events were well-characterized gastrointestinal and hematologic toxicities of this drug class and were manageable with the pre-specified dose modifications.

Overall, the PK and safety profiles were generally consistent with those of individual study drugs, supporting the proposed dose of ipatasertib 300 mg when coadministered with palbociclib 125 mg and fulvestrant. As part of the ongoing effort for dose optimization, DDI is an important aspect to consider, especially for combined treatments. Data from *in vitro* studies, clinical studies and PBPK modeling can be leveraged to evaluate the most appropriate dosing regimen to be tested to minimize subtherapeutic or supratherapeutic exposures in patients.

## Data Availability

Qualified researchers may request access to individual patient level data through the clinical study data request platform (https://vivli.org/). Further details on Roche’s criteria for eligible studies are available here (https://vivli.org/members/ourmembers/). For further details on Roche’s Global Policy on the Sharing of Clinical Information and how to request access to related clinical study documents, see here (https://www.roche.com/research_and_development/who_we_are_how_we_work/clinical_trials/our_commitment_to_data_sharing.html).
